# Analysis of a Hybrid Spine Fixation Approach for the Treatment of Unstable Thoracolumbar Fractures

**DOI:** 10.7759/cureus.31953

**Published:** 2022-11-27

**Authors:** Sanjay Yadav, Saurabh Singh, Abhinav A Jha

**Affiliations:** 1 Orthopedics, Institute of Management Studies, Banaras Hindu University (IMS BHU), Varanasi, IND

**Keywords:** decompression, fixation, hybrid, fracture, thoracolumbar, spine

## Abstract

Introduction

The treatment of unstable thoracolumbar burst fractures and fracture dislocations of the thoracolumbar spine remains ever evolving. Anterior or posterior approaches both have equal efficacy, but the posterior approach has been preferred in our study due to its ease of application, less extensile nature, and reduced intra-operative bleeding. Posterior approaches can employ short-segment fixation or long-segment fixation techniques. Long segment fixation may need implant removal later to increase mobility in nonfusion surgeries. The thoracolumbar segment is a transition zone where the thoracic spine is a less flexible zone, and the lumbar spine is a more flexible zone. Lumbar motion is important to preserve. Hence, we proposed to study spinal fixation two levels above and one level below the fracture for stabilization. This may provide increased stability along with preservation of the motion segment at the lumbar level.

Methods

We retro-prospectively reviewed the results of unstable thoracolumbar junction fractures with incomplete or intact neurology in 34 consecutive cases operated with alternate two above and one below fixation approach between June 2018 and June 2019 at our institute. Five cases were excluded due to incomplete follow up and the remaining 29 patients were included in the study. Regular follow-up in the postoperative period at three, six, and 12 months was conducted. Data analysis was done by SPSS software version 22 (IBM Corp., Armonk, NY).

Results

Twenty-nine patients were included in the study out of which 16 were males and 13 were females. The average age was 36.31±1.46 years (range, 14-60 years). The average follow-up duration was 14.31 months. The average injury to surgery interval was 7.17±7.31 days (range, 1-30 days). On analysis via paired t-test, pre-operative kyphotic angle (mean=20.06±8.34º) improved to immediate post-operative (mean=8.44±5.76º, p=0.0001). The postoperative kyphotic angle at 12 months follow-up showed significant stability (Mean=14.13±5.27º, p=0.0001). A median average pre-operative neurological compromise was ASIA score C and Frankel Grade C and the median average disability was an ODI score of 61%-80%. At the end of 12 months of follow-up the median average neurological compromise improved to ASIA Score D and Frankel Grade D and the median average disability improved to an ODI score of 21%-40%.

Conclusion

Two levels above and one level below hybrid pedicle screw fixation with decompression for the treatment of unstable thoracolumbar fractures with partial and intact neurology was successful within the limited time frame we had for follow-up in preserving progressive post-operative kyphosis, preserving one-motion segment, improving the neurological outcome and disability of the patients without any major complications.

## Introduction

The thoracolumbar level is one of the most common sites of spinal injuries. They are usually the result of high-energy trauma like road traffic accidents and falls. These thoracolumbar junctional injuries account for approximately 90% of all spinal fractures. Burst fractures with neurological injuries account for 10%-20% of thoracolumbar injuries. Failure to stabilize the spine can result in progressive deformity and prolonged immobilization with complications [1].

The management of unstable thoracolumbar injuries is constantly evolving with a better understanding of the problem. Various classification systems exist based on the mechanism of injury, fracture morphology and neurological involvement [2]. Different studies have shown that when there is a loss of >50% of the vertebral body height or kyphotic angle >20°, there exists spinal instability and the spinal segment will ultimately give away with weight bearing [3]. The goals of treatment in such fractures are reinstating the column stability, achieving spinal canal decompression and preserving lumbar mobility leading to early mobilization and prevention of postoperative kyphosis [4].

The pedicle screw system helps in instant stable fixation because the screws hold all three spinal columns. Short-segment fixation versus long-segment fixation has been a topic of debate. Short segment fixation- one up and one below has been associated with high rates of collapse in fixation and kyphosis whereas long segment fixation is associated with increased load on lower discs [5]. Fixation of the fractured/index vertebra has also been done to increase stability and prevent local kyphosis of the fractured vertebra, but it poses problems if the anterior approach is required subsequently [6].

Another tactic at the thoracolumbar region includes modified fixation with two-up and one-below stabilization below the fractured vertebra. This is suggested to provide better stability with less implant failure rates and preserve the motion segment below the fracture level. There are limited studies using this approach. Anterior or posterior approaches are both effective, but we chose the posterior approach in our study due to its ease of application, less extensile nature and reduced intra-operative bleeding [7].

Considering all these factors, we employed a hybrid fixation strategy with two-level up and one-down (2U/1D) stabilization via a posterior-only approach. The rationale was to study the effectiveness of pedicle screw fixation with such a hybrid method (two up-one down; 2U/1D) across the involved vertebra in reducing postoperative deformity progression and implant failure.

## Materials and methods

We retro-prospectively studied consecutive cases of unstable thoracolumbar fractures having partial neurological deficit operated from 2018 to 2019 in the orthopedics department of a university-level teaching hospital. The patients were treated with a hybrid fixation method with 2U/1D (2 above-1 below level) pedicle screw fixation. Institutional review board approval was obtained (IRC/IMS/1434). Written and informed consent was taken from all subjects. This study was part of the dissertation. the patients were recruited within the time frame but followed for a longer duration.

Patients aged 18-65 years with unstable thoracolumbar injury at a single level (between T10 and L3), kyphosis >20°, loss of vertebral body height >50%, minimum follow-up duration of one year and providing consent for the study were included. Exclusion criteria were patients aged <18 or >65 years, more than one level of spine injury, pathological fractures, fracture-dislocations and those not willing to participate in the study.

A total of 34 cases were operated. Three patients opted out citing personal reasons and 2 patients deceased due to unrelated causes before completing the designated follow-up. Finally, 29 patients were analyzed. Demographic data like age and gender, occupation, and mode of injury were collected. Pre-operative plain radiographs and an MRI spine were done along with a routine hematological workup. Clinical and radiological parameters- neurological assessment via ASIA score/ Frankel grading, kyphotic angle by Cobb’s method, spinal canal occlusion by the retropulsed bony fragment, fracture classification, functional assessment by Oswestry Disability Index were noted. Data were collected preoperatively, postoperatively (within two weeks) and at three, six and 12 months period using designed proforma independently by two observers (one resident and one senior author). At the final follow-up, MRI was done to assess the decompression and status of the spinal cord at the fracture site.

The operative procedure involved a posterior midline approach under general anesthesia. Following standard exposure, 2U/1D pedicle screw fixation was performed. The indirect reduction was done using ligamentotaxis. If the posterior bony fragment was not reduced on the lateral image, decompression and fusion were done at the fractured level. Post-operatively intravenous antibiotics were given for four days. The drain was removed on day 2 when the flow was reduced to 30 mL/8hr. Side turning and in-bed mobilization were allowed as soon as tolerated. Sitting and ambulation were allowed with the brace after suture removal at two weeks. Regular follow-up was done and VAS and ODI scores were noted. Plain radiographs were obtained to assess the union and implant position. MRI was done at one year to assess cord decompression.

Statistical analysis was performed using SPSS v22 from IBM Corporation (Armonk, NY). Descriptive statistics were calculated for demographic parameters. Differences between the baseline and follow-up time points in Kyphosis angle, ASIA score, Frankel grade and ODI score were analyzed by paired t-test and ANOVA. For categorical data comparison, the Chi-square test was used. The correlation among variables was evaluated by Pearson’s two-tailed bivariate analysis.

## Results

**Figure 1:**
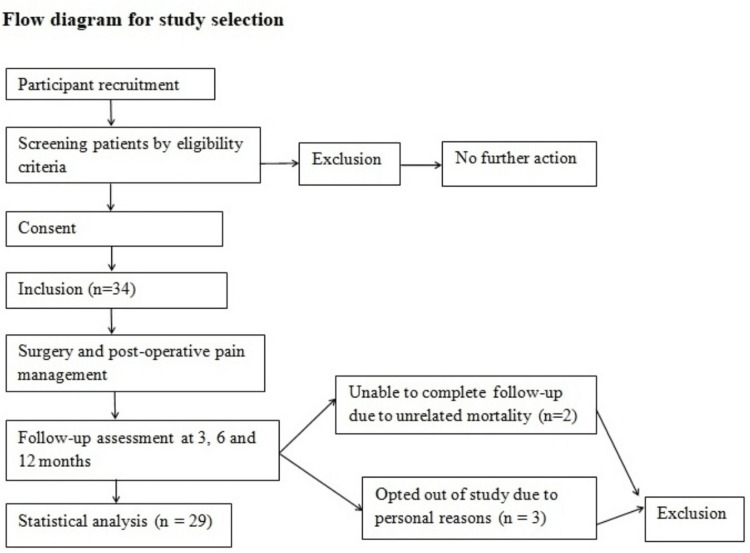
Flow diagram for the study of patient selection.

The flow of patients and final recruitment is shown in Figure 1.

Out of 29 patients analyzed, 16(55%) were males and 13(45%) were females with average age of 36.3±1.4years (range, 14-60 years). Average follow-up duration was 14.3±2.1months (min:12mo; max:23mo). Among them 11 were housewives, seven laborers, six students and five businessmen.

The most common involved level was L1 (44.8%) followed by D12 (31%), L2 (20.7%) and L3 (3.4%) cases. Twenty-four patients (82.7%) fell from height, three (10.3%) had motor vehicle accident and two (6.9%) got injured due to heavy object falling on the body. Average injury to surgery interval was 5.1±2.3 days (range, 1-14days).

Fractures were classified based on two classification systems namely McAfee and AO Spine thoracolumbar classification system. Burst type was most common as per McAfee classification and posterior tension band injuries along with burst type were most common according to AO spine classification system.

Average pre-operative kyphotic angle was 20.1 ± 8.3º which improved to 8.4 ± 5.7º in the immediate post-operative period which gradually increased to 10.3 ± 5.6º at three months, 11.7 ± 5.3º at six months and 14.1 ± 5.2º at one-year interval. The kyphotic angle improvement was statistically significant post-operatively (p<0.001) and at one-year follow-up (p<0.001).

**Figure 2:**
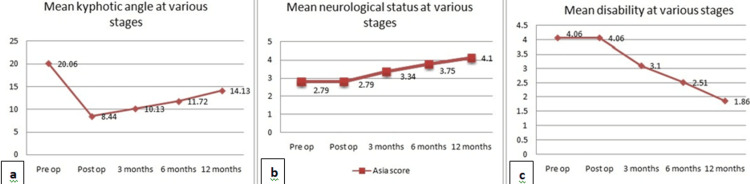
Graphs showing (a) mean kyphotic angle at various stages of follow up; (b) mean neurological status at various stages of follow-up; (c) mean disability at various stages of follow-up.

Median pre-operative neurological compromise was ASIA score C and Frankel Grade C and median average disability was ODI score of 61%-80%. Post-operatively, there was no significant improvement in these values. At the end of 12 months, median neurological compromise improved to ASIA Score D and Frankel Grade D. Median disability improved to ODI score of 21%-40% (Figures 2a-2c).

**Figure 3:**
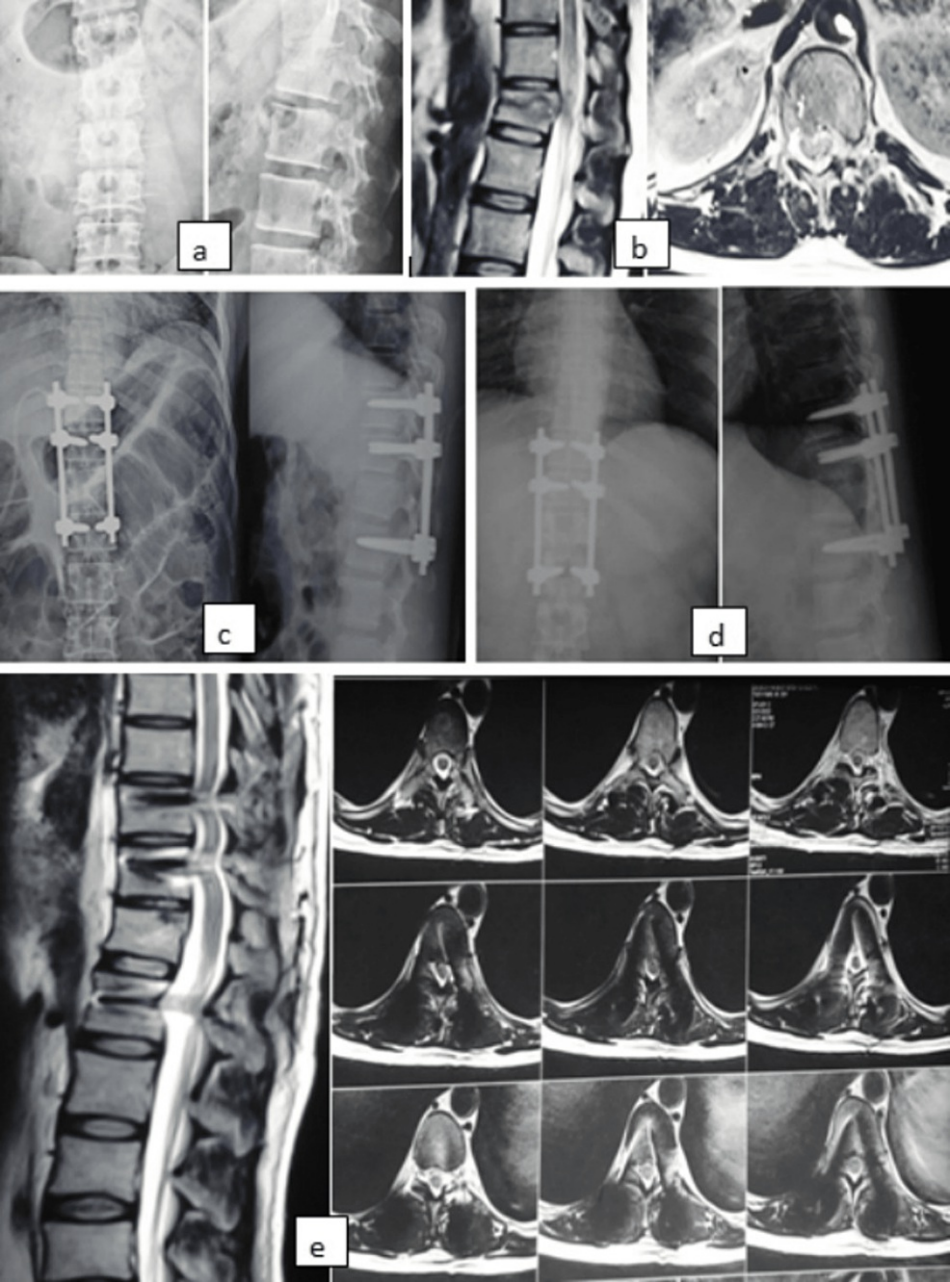
Case example showing (a) preoperative x-ray AP and lateral view showing D12 vertebra fracture with kyphosis angle of 18ᵒ. (b) Preoperative MRI showing burst fracture of D12 vertebra with cord edema and spinal canal occlusion of 20% and 30% in sagittal and axial view, respectively. (c) Immediate post-operative radiographs (kyphosis - 4ᵒ) and (d) final follow-up radiographs at 12 months (kyphosis - 8ᵒ) showing correction of kyphosis and maintenance. (e) MRI at 12 months follow-up showing restoration of vertebral height and resolution of cord edema and spinal canal occlusion.

In MRI sagittal section average occlusion of the spinal canal was found to be 47% and in axial section the average occlusion was 56%. Indication for surgical stabilization was based on the Thoracolumbar Injury Classification and Severity (TLICS) score. Based on this, 27 patients had TLICS score ≥5 and two patients had score 4. Out of 29 patients, 22 patients had posterior ligamentous complex (PLC) damage as correlated intra-operatively. Direct decompression with laminectomy was performed in 12 patients. One case example is shown in Figures 3a-3e.

Pearson’s two- tailed bivariate (r) analysis was performed to find out the correlation among the variables. It was found that age was correlated to pre-operative kyphotic angle (r = -0.49), preoperative ODI score (r = -0.36), preoperative ASIA score and Frankel grade (r = -0.37) and ASIA score and Frankel grade at one-year follow-up (r = 0.43) which meant younger patients had greater pre-operative kyphotic angle, but better neurological status and disability as compared to older patients involved in the study. Gender of the patient was correlated to the level of injury (r = 0.38) suggesting that males sustained injury at the lower level of thoracolumbar junction whereas females at the upper level.

Socioeconomic status (Kuppuswamy scale) of the patient played an important part in determining the surgical outcome of the patient during the follow-ups. It was correlated to pre-operative kyphotic angle (r = 0.42), ODI score at 12mo (r = 0.48), preoperative ASIA score (r = -0.36) and ASIA score at 12mo (r = -0.45). Above findings suggest that people from lower socioeconomic class had higher pre-operative kyphotic angle and slower recovery post-surgery.

Spinal canal occlusion in MRI sagittal view corresponded to pre-operative ODI score (r = 0.52), ODI score at 12mo (r = 0.45), pre-operative ASIA score (r = -0.36), ASIA score at 12mo (r = -0.39), McAfee fracture type (r = -0.53), AO spine thoracolumbar fracture type (r = 0.51) and laminectomy status (r = -0.39). All the above data basically suggests that greater the occlusion of the spinal canal due to the retropulsed bony fragments poorer the pre-operative neurological and disability status, increased tendency for intra-operative laminectomy and delay in recovery of the patients.

## Discussion

The best possible surgical management for unstable thoracolumbar fractures is still controversial. The evidence-based guidelines or instrumentation techniques based on the most suitable surgical approach for unstable thoracolumbar fractures with incomplete neurological deficits are still lacking. However, posterior transpedicular screw fixation is used commonly in routine practice due to its low morbidity, less extensile nature, controlled blood loss and a surgical field devoid of major neurovascular structures. Due to these reasons, we opted for posterior transpedicular fixation for the management of such unstable thoracolumbar fractures in our study [8].

Posterior transpedicular fixation was first mentioned by Boucher in 1959 [9]. It provides stability, maintains reduction until the bony union is achieved, preserves motion at segments and avoids long segment fusions. Short-segment pedicle fixation had a high failure rate due to progressive kyphosis because of screw bending, bony collapse or vertebral body translation. Four pairs of screws (two above-two below) to increase the lever arm enhancing the stability and better reduction of the kyphotic deformity were also proposed. Long-segment fixation is an effective way to treat thoracolumbar fractures but has the disadvantage of prolonged operative time and blood loss [10]. McAfee et al. reported that two levels above and below the injured level in an unstable calf spine model were stiffer than the intact spine [11].

Carl et al. reported that segmental transpedicular fixation two levels above the kyphosis should be used at the thoracolumbar junction because the compressive forces act more anterior. The fixation one level distal to the fracture might prevent the motion segment in the lumbar spine where compressive forces act more posteriorly [12]. Therefore, intermediate and long posterior segment fixation seems to be an effective way of managing unstable thoracolumbar fractures.

Our study is a single-center work that increases the possibilities of standardized selection of patients and surgical technique. It is a prospective observational cohort study. Many studies have been conducted demonstrating the biomechanics of the construct used, comparison between the short and long segment fixation and complications of the various surgical techniques but in our study, we have tried to reveal other aspects which include socioeconomic aspects, neurological outcomes, the status of low back pain, intraoperative and radiological findings related to its surgical management with two levels up and one down posterior fixation of unstable thoracolumbar fractures at the end of 12mo follow-up.

The most common injury mechanisms in our study were fall from height and motor vehicle accidents. Most fractures in the present study occurred at the L1 level, females tend to have fractures at the upper part of the thoracolumbar junction whereas males tend to have fractures at the lower part. Alanay et al. reported T12 as the most common injured level [13]. Farcy et al. noted L2 in males and L1 in females to be the most commonly affected vertebra. Males are more susceptible to thoracolumbar fractures than females because they are involved in outdoor activities to a larger extent [14].

Males were 55.2% and females were 44.8%. Most cases were from the upper middle class according to the Modified Kuppuswamy scale. We observed that with the lowering of socioeconomic class, the preoperative kyphotic angle increased, and disability and neurological recovery also got delayed which may be due to the work-related injury in this group. Spinal trauma was relatively common in younger age groups with a higher preoperative kyphotic angle, but preoperative disability and neurological deterioration were less severe compared to the older age group.

In our study, 44.8% were burst fractures and 48.3% were due to posterior tension band injury according to AO Spine Thoracolumbar classification, and 89.7% were burst type according to McAfee classification. Kim et al. in their work found 50% of thoracolumbar fractures to be of burst type [15].

Furthermore, we observed that patients with burst fractures had an increased tendency of being crippled/bedbound at the preoperative stage and had increased spinal canal occlusion as observed in MRI as compared to other fracture subtypes. Kumar et al. suggested that the injury to surgery interval is important as it determines the efficacy of ligamentotaxis [16]. The efficacy of the ligamentotaxis has been shown to decrease after traumatic injury within 72 hours. In our study, 72.4% of the patients were operated on within seven days post-trauma. We agree with the fact that earlier the intervention was done better for the correction of the kyphotic angle post-surgery.

Modi et al. [17] in a similar study mentioned the average preoperative kyphotic angle of 26.7° which recovered to 4.1° after surgery and maintained at 6.3° at the final follow-up. Our study showed similar outcomes at preoperative and postoperative stages but there was a gradual rise in the kyphotic angle at the final follow up which suggests a steady rise in kyphotic angle as patients starts weight bearing and Modi et al. have mentioned that laminectomy was not performed in their study group, but a significant number of patients underwent laminectomy in our case; therefore, we can explain the gradual increase in kyphosis at the final follow-up.

Our result showed improved outcomes as compared to short segment fixation in maintaining progressive kyphosis as reported in the literature. We agree with the fact that instrumenting the fracture two levels above and one below prevents progressive kyphosis development. Additionally, we also evaluated the low back pain and neurological status of the patient in the study group. Most patients who were previously bed-bound or crippled were mobilized at the final follow-up or in a lesser time.

Butt et al. [18] reported the success of short-segment fixation in thoracolumbar burst fractures. However, 20 out of 50 patients had a hardware failure (40%) but in our study, no hardware failure was documented at the end of one year of follow-up. The strength of construct was strong and did not lead to implant failure and was successful in preserving the motion segment at the lumbar level. Implant failure which is a common problem in short-segment fixation has not been encountered so far in our study and at the same time, we have also avoided anterior fixation and long-segment fixation which have their drawbacks.

There were certain limitations like having limited samples for data analysis and follow-up duration. Mid to long-term follow-up may be more beneficial in identifying long-term problems and complications as the continuation of this work. A randomized controlled trial comparing two up-one down hybrid pedicle fixations with standard short segment one up-one down fixation in such fractures can help in providing more evidence to the work.

## Conclusions

The study discusses an alternate “hybrid” spinal fixation strategy to preserve lumbar motion. We conclude that the two level up and one below-pedicle screw fixation approach with spinal decompression is a rational alternative to managing unstable thoracolumbar fractures with incomplete neurological deficits. It prevents progressive kyphosis development, preserves one motion segment at the lumbar region and supports early mobilization. However, long-term follow-up is needed.
